# Narrow Genetic Diversity of *Wolbachia* Symbionts in Acrididae Grasshopper Hosts (Insecta, Orthoptera)

**DOI:** 10.3390/ijms23020853

**Published:** 2022-01-13

**Authors:** Yury Ilinsky, Mary Demenkova, Roman Bykov, Alexander Bugrov

**Affiliations:** 1 Laboratory of Molecular Genetics of Insects, Institute of Cytology and Genetics SB RAS, 630090 Novosibirsk, Russia; judina@bionet.nsc.ru (M.D.); bykovra@bionet.nsc.ru (R.B.); 2 Center for Mitochondrial Functional Genomics, Immanuel Kant Baltic Federal University, 236041 Kaliningrad, Russia; 3 Faculty of Natural Sciences, Novosibirsk State University, 630090 Novosibirsk, Russia; bugrov04@yahoo.co.uk; 4 Institute of Systematics and Ecology of Animals SB RAS, 630091 Novosibirsk, Russia

**Keywords:** Acrididae, horizontal transmission, multilocus sequence typing, recombination, population, symbiont, *Wolbachia*

## Abstract

Bacteria of the *Wolbachia* genus are maternally inherited symbionts of Nematoda and numerous Arthropoda hosts. There are approximately 20 lineages of *Wolbachia*, which are called supergroups, and they are designated alphabetically. *Wolbachia* strains of the supergroups A and B are predominant in arthropods, especially in insects, and supergroup F seems to rank third. Host taxa have been studied very unevenly for *Wolbachia* symbionts, and here, we turn to one of largely unexplored insect families: Acrididae. On the basis of five genes subject to multilocus sequence typing, we investigated the incidence and genetic diversity of *Wolbachia* in 41 species belonging three subfamilies (Gomphocerinae, Oedipodinae, and Podisminae) collected in Turkey, Kazakhstan, Tajikistan, Russia, and Japan, making 501 specimens in total. Our results revealed a high incidence and very narrow genetic diversity of *Wolbachia*. Although only the strains belonging to supergroups A and B are commonly present in present, the Acrididae hosts here proved to be infected with supergroups B and F without A-supergroup variants. The only trace of an A-supergroup lineage was noted in one case of an inter-supergroup recombinant haplotype, where the *ftsZ* gene came from supergroup A, and the others from supergroup B. Variation in the *Wolbachia* haplotypes in Acrididae hosts within supergroups B and F was extremely low. A comprehensive genetic analysis of *Wolbachia* diversity confirmed specific features of the *Wolbachia* allelic set in Acrididae hosts. This result can help to elucidate the crucial issue of *Wolbachia* biology: the route(s) and mechanism(s) of *Wolbachia* horizontal transmission.

## 1. Introduction

Bacteria of the *Wolbachia* genus are maternally inherited symbionts of Nematoda and numerous Arthropoda hosts. The wide distribution of *Wolbachia* in insect hosts resembles a pandemic [1]. To spread through a host population, the *Wolbachia* symbionts induce reproductive abnormalities such as cytoplasmic incompatibility, parthenogenesis, male killing, and feminisation [2]. Additionally, there is evidence of mutualistic effects between *Wolbachia* symbionts and their hosts [3]. There are ~20 lineages of *Wolbachia* called supergroups, and these are designated alphabetically with some omissions [4,5,6]. Strains belonging to supergroups A and B are predominant in arthropods, especially in insects, and the F supergroup seems to take third place and is partitularly detectable in Nematoda [7], Coleoptera [8,9,10], Diptera [11], Hemiptera [12], Hymenoptera [1,13,14], Isopoda [15], Odonata [16], Scorpiones [17], Strepsiptera [18], and Termites [1,19,20,21]. Strains of other *Wolbachia* lineages are found much less frequently and are often associated with a specific host taxon. Similar or even the same *Wolbachia* strains can be found in unrelated hosts, implying the horizontal transmission of the symbiont. The routes of interspecies transmission are not well understood at present. Predation, host–parasitoid interactions, and feeding on a common substrate are possible mechanisms [22,23,24,25,26,27,28,29,30,31]; however, the main transmission pathway remains unknown.

Acrididae grasshoppers comprise more than 10,000 species that are widespread in nearly all terrestrial landscapes. Due to the great importance of Acrididae grasshoppers as agricultural pests, much research attention has been given to this taxon.

Data on *Wolbachia* in Acrididae are fragmentary. *Podisma pedestris* [32] and *Podisma sapporensis* [33] are known to be affected by *Wolbachia* infection. Samples of *Acrida willemsei*, *Calliptamus italicus*, *Ceracris fasciata*, *Catantops humilis*, *Chorthippus brunneus*, *Hieroglyphus banian*, *Melanoplus* sp., *Oxya ntricata*, and *Oxya japonica* have been reported to be uninfected [23,34,35,36,37]. A series of studies have been performed on populations of *Chorthippus parallelus* inhabiting French and Spanish territories [38,39,40,41,42,43,44,45], where investigators have reported *Wolbachia* strains of supergroups B and F and even B/F recombinants.

Here, we evaluated the genetic diversity of *Wolbachia* in members of the Acrididae family. In particular, we screened specimens from three subfamilies (Gomphocerinae, Oedipodinae, and Podisminae) collected in Turkey, Kazakhstan, Tajikistan, Russia, and Japan and characterised *Wolbachia* isolates by a multilocus sequence typing (MLST) protocol that included five housekeeping genes [12]. We aimed to investigate *Wolbachia* incidence in Acrididae hosts and to determine whether there is a *Wolbachia* genetic pattern that is specific to Acrididae.

## 2. Results

### 2.1. Wolbachia Occurrence in Acrididae

We examined the infection status in 41 Acrididae species and found 28 *Wolbachia*-infected species (Table 1). Only two species have been analysed previously: *P. sapporensis* [33] and *P. pedestris* [32]. The symbionts were found in all of the subfamilies under study: Gomphocerinae, Oedipodinae, and Podisminae. There were no noticeable differences in *Wolbachia* prevalence between the regions and the between specimen collection years. The estimation of infection prevalence was not the aim of our study because we mainly tested somatic tissues that may only reflect the lowest rate boundary. Nevertheless, we registered high *Wolbachia* prevalence in *Ch. biguttulus* (174/198), *Ch. fallax* (16/17), and *P. montanus* (40/50). High *Wolbachia* prevalence in *P. sapporensis* populations was determined based on gonad tissues and was reported earlier [33]; here, we screened an additional 18 specimens for infection and confirmed the previous conclusion. Sample sizes were not sufficient to make firm conclusions about other species. 

### 2.2. Genetic Diversity of Wolbachia Isolates

A total of 44 *Wolbachia* isolates were characterised by the MLST protocol [12]; 43 were unique according to the species–site–year combination (Table 2). We managed to amplify all five loci for 39 isolates, and for 35 isolates, we obtained unambiguous allele sequences. For many isolates, there were difficulties in obtaining complete MLST profiles. In two cases (*ftsZ* of i-1 and *gatB* of i-15), we failed to obtain amplicons, even when using nested PCR. For several isolates, we could not obtain good quality sequences; there were double chromatogram peaks for certain positions or loci, or in some cases, even repeated double peaks in the bulk of a sequence. 

**Figure 1:**
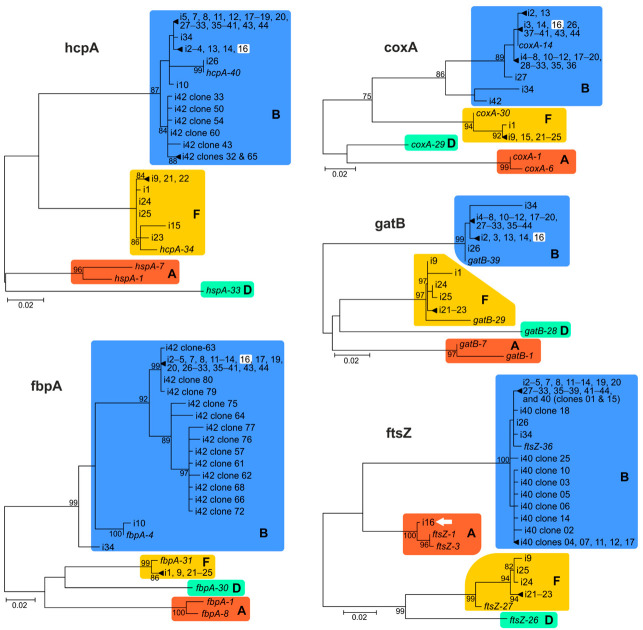
Maximum likelihood (ML) phylogenetic trees for each MLST gene. Acrididae isolates (Table 2), supergroups (A, B, F, and D), and bootstrap values are indicated. Model of nucleotide substitutions T92+G was used for *gatB*, *hcpA*, and *fbpA* datasets; HKY+G for *coxA*; T92+G for *ftsZ*. White squares and the arrow indicate a case (i-16) of a supergroup clustering conflict. See original files in Supplementary Materials.

**Figure 2:**
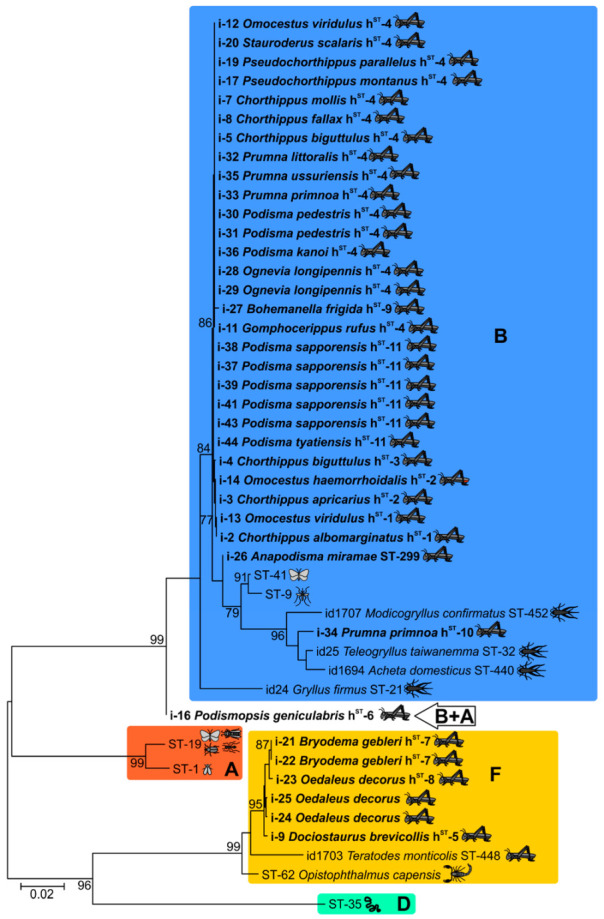
An ML phylogenetic tree of *Wolbachia* symbionts isolated from Acrididae hosts. The dataset is based on the concatenation of five genes of the MLST protocol, model T92+G, and bootstrapping with 1000 iterations (values higher than 75 are provided). See original files in Supplementary Materials.

We reconstructed ML phylogenetic trees for each locus to examine supergroup clustering (Figure 1, Supplementary Materials). Alleles of the analysed isolates belong to *Wolbachia* supergroups B and F. In particular, species belonging to Podisminae only harboured variants from supergroup B, species belonging to Oedipodinae only harboured variants from supergroup F, and species belonging to Gomphocerinae mostly harboured species from super group B with some from supergroup F. Overall, the genetic diversity of the MLST loci appeared rather low (Table 2; Figure 1 and Figure 2, Supplementary Materials). This result was especially evident at the *fbpA* locus, where nearly all of the B-supergroup isolates contained the *fbpA-197* allele and where all of the F-supergroup isolates contained *fbpA-410*. Moreover, there was one case of an inter-supergroup recombination. A complete haplotype of i-16 (*Podismopsis genicularibus*) was found to be basal to supergroup B because the *ftsZ-226* allele belonged to supergroup A, whereas alleles from other loci were assigned to supergroup B. 

Only haplotype ST-299 has been reported upon earlier [46], whereas others turned out to be unique because of new alleles or new allele combinations. A new combination of previously known alleles (*gatB-9*, *coxA-133*, *hcpA-6*, *ftsZ-106*, and *fbpA-197*) that we designated as ST h^ST^-4 (see Materials and Methods) was the most frequent in the study population; it was present in seven species of Gomphocerinae and in six species of Podisminae. Haplotypes h^ST^-1, -2, -3, -9, and -11, which are closely related to h^ST^-4 (*p*-distance 0.0005–0.0020, namely, 1–4 mutations), were also revealed in Gomphocerinae and Podisminae species in geographically distant populations. These six haplotypes form an ‘h^ST^-4 group’ that includes a constant allele set of *ftsZ-106* and *fbpA-197* as well as varied but closely related (to each other) alleles of *gatB*, *coxA*, and *hcpA* loci. Other B-supergroup haplotypes were distantly related to h^ST^-4 (*p*-distance for ST-299: 0.0058, for h^ST^-6: 0.0203 and for h^ST^-10: 0.0323); however, they shared identical alleles with the h^ST^-4 group. These phenomena can be explained by intra-supergroup recombination.

*Wolbachia* strains belonging to supergroup F were detected in five species. Haplotypes of complete profiles (h^ST^-5, -7, and -8; *p*-distance 0.0024–0.0053) and isolates with incomplete profiles (i-1 and i-15) or with ambiguous sites (i-24 and i-25) were found to be closely related, i.e., genetic diversity was also rather low. A comparative analysis of the haplotype diversity of supergroup F retrieved from the PubMLST database showed that variants of the Acrididae isolates formed a separate cluster (Figure S1).

**Figure 3:**
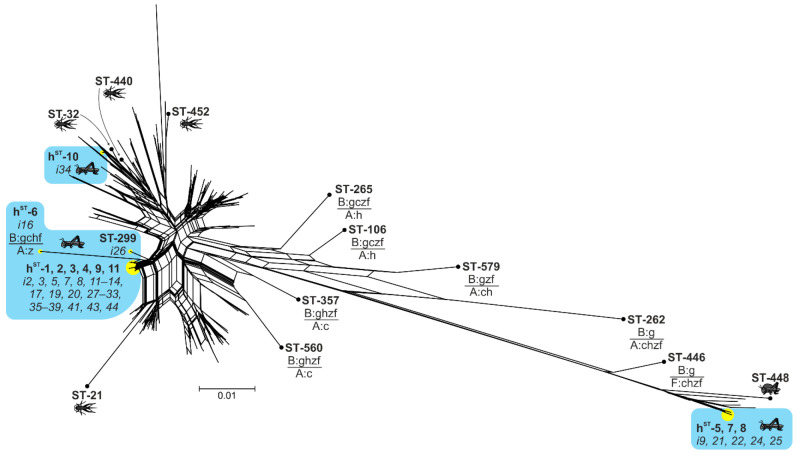
The phylogenetic network (NeighborNet) constructed in SplitsTree4 from 215 unique haplotypes (concatenated MLST genes). Branches with isolates from this study are highlighted in yellow. Other haplotype profiles were included here because (1) an Orthoptera host (insect symbol provided) or (2) a haplotype shared at least one identical or closely related allele with the studied Acrididae isolates. Inter-supergroup recombinant haplotypes are designated as ‘supergroup (A, B or F): genes g (gatB), c (coxA), h (hcpA), z (ftsZ), f (fbpA)’. See original files in Supplementary Materials.

To examine the relationship of the *Wolbachia* variants isolated from Acrididae with other *Wolbachia* strains, we reconstructed a phylogenetic network (Figure 3, Supplementary Materials). The dataset that was used for this analysis included *Wolbachia* haplotypes from different hosts that contained at least one allele that was identical or that was closely related to *Wolbachia* isolates from Acrididae (see Materials and Methods). Most *Wolbachia* isolates of Acrididae (actually the h^ST^-4 group) formed a separate bundle in the phylogenetic network without isolates from other hosts. Even i-16, which is an inter-supergroup recombinant, occupied a long branch in this bundle. The remaining *Wolbachia* haplotype h^ST^-10 (i-34) manifested a close relationship with isolates from Gryllidae hosts. Of note, i-34 was isolated from the *Prumna primnoa* population of Sakhalin in 2010, where the h^ST^-4 (i-33) haplotype was identified as well. Haplotypes of the F supergroup also formed a separate bundle. Only the F-supergroup *Wolbachia* haplotype ST-448 isolated from *Teratodes monticollis* (Acrididae: Teratodinae) was noticeably different in the allele set and genetic distance (*p*-distance 0.0293–0.0313). Therefore, the additional data from the MLST profiles based on allele similarity confirmed that the analysed Acrididae subfamilies have a specific genetic pattern of *Wolbachia*.

**Figure 4:**
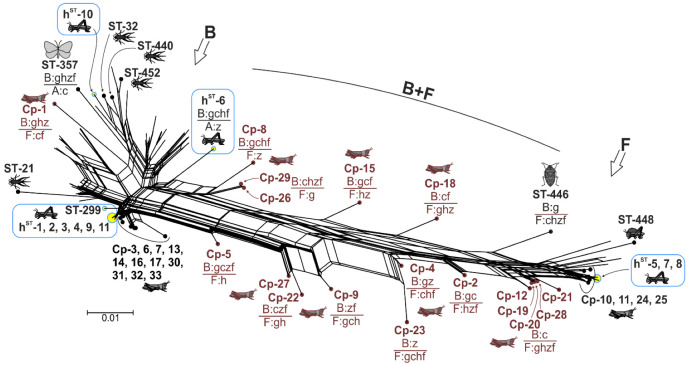
The phylogenetic network (NeighborNet) constructed in SplitsTree4 from 127 unique haplotypes (concatenated MLST genes). Branches with isolates from this study are highlighted in yellow, isolates from [44] (Cp) are in brown. The dataset of this figure differs from that in Figure 3 in the following ways: (1) the number of haplotypes was reduced (to decrease phylogenetic noise), (2) the alignment corresponds to haplotype data from [44]. Inter-supergroup recombinant haplotypes are designated as in Figure 3. See original files in Supplementary Materials.

Martinez-Rodriguez and Bella [44] reported the *Wolbachia* MLST diversity of *Ch. parallelus* in Spanish and French locations. We retrieved sequences of five MLST loci deposited by [44] in GenBank, combined them with the dataset of our phylogenetic network, excluded redundant portions of sequences, and reconstructed a new phylogenetic network (Figure 4, Supplementary Materials). Half of the *Wolbachia* variants from *Ch. parallelus* matched the genetic pattern discovered in our collection for both the B- and F-supergroup haplotypes. The other half represented B–F supergroup recombinants. B–F recombinants were registered by [44]; here, we reported detailed characteristics of some variants (details in Figure 4). It is worth pointing out that the recombination occurred at all possible loci, thereby giving rise to 18 unique haplotypes in total.

### 2.3. Incomplete MLST Profiles

As mentioned above, for some isolates, we could not obtain an amplicon or unambiguous sequences. Amplicons of three such cases in two isolates of *P. sapporensis* (i-40 and i-42) were cloned and sequenced. Fifteen clones of an *ftsZ* amplicon of i-40 represented 11 variants; among them, we regard two variants—*ftsZ-106* (two clones) and *ftsZ-81* with substitution A^408^ (five clones)—as authentic (see the last sentence of this subsection) because the former is commonly found in other *P. sapporensis* isolates, and the latter one was assembled manually after direct amplicon sequencing. The other eight variants represented by unique clones were close to the alleles *ftsZ-7*, *-81*, and *-106*, but they differed by amino acid substitutions, and there was even a stop codon in clone 5. A similar pattern was observed during the screening of the *hcpA* and *fbpA* amplicons of i-42. There were seven variants that were closely related to the allele *hcpA-142* with 4–7 mutations, including the variant *hcpA-142* G^298^ A^373^ G^393^ T^438^, which was manually assembled after direct amplicon sequencing. In the case of *fbpA* cloning, one variant matched *fbpA-197*, two differed by one mutation, and the others differed by 13–19 mutations. Therefore, the cloning of the PCR products revealed several variants: *ftsZ*-*106*, *ftsZ*-*81* A^408^, and *hcpA-142* G^298^ A^373^ G^393^ T^438^ in i-40 as well as *fbpA*-*197* and probably one mutant variant of *fbpA*-*197* in i-42. They most likely characterise the *Wolbachia* genome, whereas other variants quite possibly reflect *Taq* polymerase artefacts or denote *Wolbachia* genome segments that became integrated into the host genome.

## 3. Discussion

The Acrididae family includes more than 10 thousand species inhabiting different climatic zones from tropical rainforests to the subarctic zone. There is extensive information about the geographical distribution and economic significance of this group of insects and about the factors affecting the regulation of grasshopper population size in natural and anthropogenic landscapes. Nevertheless, knowledge about the symbionts of this group of insects remains meagre. Here, we presented the most detailed survey of *Wolbachia* diversity in Acrididae to date; 28 species out of 40 appeared to be infected by *Wolbachia*. On the other hand, this list, together with other studies [23,32,33,34,35,36,37,44], comprises less than 1% of known Acrididae species. To achieve a more informative picture of *Wolbachia* genetic diversity in this family, it is necessary to examine other big subfamilies, and special attention should be paid to collections from warm climate zones, where the diversity is especially high. Currently, our results indicate that *Wolbachia* infection (i) is widespread among three subfamilies of Acrididae grasshoppers, (ii) can reach high prevalence in populations, and (iii) can be detected by means of DNA isolated from somatic tissues. 

Our main result is the narrow genetic diversity of *Wolbachia* variants in Acrididae hosts. While *Wolbachia* in insects is commonly represented by the strains of supergroups A and B, the Acrididae hosts proved to be infected with supergroups B and F without A-supergroup variants. The only trace of an A-supergroup lineage was found in i-16, where the haplotype h^ST^-5 includes the *ftsZ* gene from supergroup A and other genes from the B supergroup. The variation of the B-supergroup haplotypes in Acrididae is especially low, as clearly illustrated by the phylogenetic network (Figure 3), where all the haplotypes included in the analysis showed allelic similarity with grasshopper *Wolbachia*. Previously, an allele set (with central haplotype ST-41) specific to butterfly hosts was characterised [46]; however, the *Wolbachia* variation observed here in Acrididae is much lower. The accumulation of data on *Wolbachia* infection in Acrididae hosts will possibly shed light on other *Wolbachia* variants, including A-supergroup variants. Nonetheless, it is already obvious that Acrididae hosts have a unique infection profile in the global pattern of the *Wolbachia* pandemic.

Active horizontal transmission (HT) of a specific set of *Wolbachia* strains among Acrididae grasshoppers is a reason for the observed narrow diversity. Here, we documented (i) cases (often seen in *Wolbachia* studies) where the same haplotype is found in different species (Table 2), (ii) findings of identical haplotype profiles in populations of *Ognevia longipennis* from Japan and Altai Mountains of Russia even though these populations most likely have been geographically isolated for several thousand years, and (iii) distantly related *Wolbachia* haplotypes within the Sakhalin population of *P. primnoa* (Sakhalin 2010). There is no clear understanding of the HT mechanism, although some data have been reported [22,25,26,29,47]. Potential vectors of *Wolbachia* HT are parasitoid wasps, red velvet mites, tachinids, entomophilic nematodes, and horsehair worms (Nematomorpha) that have wider or narrower specificity to Acrididae hosts. The next question is the nature of *Wolbachia* specificity to hosts. Are Acrididae species only susceptible to certain variants of *Wolbachia*, or does the susceptibility extend to all encountered variants?

The *Wolbachia* allelic diversity observed in our study is consistent with the findings about *Wolbachia* in *Ch. parallelus* studied on the Perinea peninsula [44]. A comprehensive analysis of the data generated by [44] and our results indicates that *Wolbachia* variants belonging to supergroups B and F are widespread in Acrididae hosts. Moreover, *Ch. parallelus* was found to harbour nearly every possible MLST recombinant combination of supergroups B and F (Figure 4). In our work, we were only able to reliably detect one isolate of inter-supergroup recombination. In five isolates, we obtained ambiguous signals in chromatograms. The nature of the problem for three of them (i-6, -15, and -18) was not investigated here, i.e., co-infection with B and F lineages cannot be ruled out. Two other isolates were cloned, and we came to the conclusion that the results can be explained by multi-infection with B-supergroup strains and/or by insertions of *Wolbachia* genes into the host’s nuclear genome. The transfer of *Wolbachia* genes into a host nuclear genome in insects has been well documented [48,49,50,51,52], e.g., in Orthoptera [42,43]. 

Another essential issue of *Wolbachia* diversity is the concept of a bacterial species [53,54,55]. Every *Wolbachia* supergroup is a species candidate [6,56,57,58,59]. This observation implies genome specificity, ecology specificity, and ‘reproductive isolation’, which in bacteria, take the form of a low rate or impossibility of gene exchange between strains of different supergroups/species. Indeed, recombination between strains of supergroups A and B is known to occur, albeit at a low rate [46,57,60,61,62,63]. Recombination between supergroups F and B has been only detected in *Chrysocoris stollii* (Hemiptera) [62]; however, in the case of *Ch. parallelus*, there are 18 haplotypes [44]. Comprehensive genomic analysis of core genes indicates that the F supergroup clusters together with C and D lineages found in nematode hosts [63,64]. The F lineage has retained the genes responsible for homologous recombination (data not shown); we concluded this after the examination of the wCle genome (GenBank accession No.: AP013028). Previously, the evolution of these genes was studied in the genomes belonging to supergroups A, B, C, and D [65] but not in F. These data suggest that gene exchange between the genomes of B and F strains in *Ch. parallelus* may occur via homologous recombination. If rampant recombination has actually occurred in *Ch. parallelus*, it casts serious doubt on the idea that supergroups B and F can be considered independent bacterial species. 

## 4. Materials and Methods

### 4.1. Collection of Specimens

Grasshopper specimens were collected from natural populations during the period of 2001–2017 (Table 1). The specimens were fixed in 96% ethanol and were stored at −20 °C. The total study population included 501 specimens from Gomphocerinae, Oedipodinae, and Podisminae, with four species being predominant (64.3%): *Chorthippus biguttulus* (215), *Chorthippus fallax* (17), *P. sapporensis* (35), and *Pseudochorthippus montanus* (50).

### 4.2. Screening and Sequencing

A leg of an individual was used for DNA extraction in most cases. This approach is rather convenient because it allows the procedure to be repeated in cases of failed extraction (just take another leg) and to avoid bacterial contamination from the digestive system due to the facultative predation/cannibalism of Orthoptera species. Nonetheless, the use of somatic tissues does not permit a reliable estimation of *Wolbachia* prevalence in a population. Male gonad tissues were used for the screening of *Podisma* species collected in Hokkaido (Japan) and Kunashir (Russia), which were partially reported by Bugrov et al. [33]. Here, we added 18 specimens and present full MLST profiles for the *Podisma* spp. hosts. DNA extraction from each sample was performed in 0.3–0.6 mL of extraction buffer (0.1 M NaCl, 10 mM Tris- HCl (pH8.0), 25 mM EDTA, 0.5% SDS, and 0.1 mg/mL proteinase K) for 2 h at +56 °C, and DNA was then salted out with 0.5 V of 5 M potassium acetate/3 M acetic acid, after which DNA was further precipitated and dissolved in 0.2 mL of double-distillated H_2_O. Next, 1 µL of the DNA solution was used in all polymerase chain reactions (PCRs). The quality of the DNA was checked with universal primers specific to the nuclear gene of 28S rRNA (28sF3633: 5′-TACCGTGAGGGAAAGTTGAAA-3′, and 28sR4076: 5′-AGACTCCTTGGTCCGTGTTT-3′ [66]) or to mitochondrial gene *CO1* (LCO1490: 5′-GGTCAACAAATCATAAAGATATTGG-3′, and HCO2198: 5′- TAAACTTCAGGGTGACCAAAAAATCA-3′ [67]). PCR was conducted using BioMaster HS-Taq PCR (2×) (BiolabMix, Novosibirsk, Russia) or a mix containing 3.0 mM Mg^2+^, 0.6 mM each primer, 1× PCR buffer (16 mM (NH_4_)_2_SO_4_, 67 mM Tris–HCl pH 8.8 (at 25 °C), and 0.1% of Tween 20), and 1.0 U of Taq polymerase in a total reaction volume of 20 μL. The detection of Wolbachia infection in each specimen was performed by means of at least two MLST loci, as usually done with primer sets coxAF1/R1 and ftsZF1/R1 [12]. All five MLST loci were amplified and sequenced for *Wolbachia*-positive DNA samples. In cases of a weak amplicon signal or negative PCR results, the nested-PCR approach was employed [68]. External primer sets F2/R2 or F3/R3 were used for *gatB*, *coxA*, *hcpA*, and *fbpA* loci according to [https://pubmlst.org/organisms/wolbachia-spp/protocol-single-infected, accessed on 12 December 2021], primers ftsZunif1/2 for the *ftsZ* locus according to [6]; and the inner primers F1/R1 according to [12]. The thermal cycling conditions were as follows: initial denaturation at 95 °C 5 min, followed by 35 cycles of conventional PCR, and 15 + 30 cycles of nested PCR at 95 °C for 15 s, annealing at 55 °C for the MLST primers or at 58 °C for 28S or at 53 °C for *CO1* for 40 s, elongation at 72 °C 30 s–1 min, and final elongation for 3 min. In the second round of nested PCR, we added 0.5 µL of the reaction mixture from the first round. The PCR products were visualised by agarose gel (1.0–1.5%) electrophoresis with ethidium bromide. A portion of the amplification reaction mixture was diluted as follows: 2 µL of the amplicon + 18 µL of water; then 1 µL of this solution was treated with 10 U of exonuclease I (New England Biolabs, Ipswich, MA, USA) in the supplied buffer and sequenced using the BrightDye Terminator Cycle Sequencing Kit (Nimagen, Nijmegen, The Netherlands) or BigDye Terminator v3.1 cycle sequencing Kit (Applied Biosystems, Foster City, CA, USA). Three amplicons (*ftsZ* of i-40; *hcpA* and *fbpA* of i-42, which yielded ambiguous sequences) were cloned in the pAL-TA vector (Evrogen, Moscow, Russia) according to the manufacturer’s instructions and were sequenced with the M13 primer set. The MLST profiles of the *Wolbachia* isolates were deposited in the GenBank database under accession numbers MZ816445–MZ816686. Because the MLST database has not been accepting new submissions for some time, here we designated haplotypes with new combinations of alleles or new alleles as ‘h^ST^-*Number*’ (Table 2).

### 4.3. Evolutionary Analysis

New MLST loci sequences were checked for stop codons, and sequence length was limited according to the MLST protocol for subsequent allele analysis. All of the sequences were used to reconstruct maximum likelihood (ML) phylogenetic trees of each MLST locus. As a supergroup reference, we used the alleles that had been retrieved from the following sequence types (STs): ST-1 (supergroup A), ST-19 (A), ST-41 (B), ST-35 (D), and ST-62 (supergroup F). Moreover, we reconstructed an ML phylogenetic tree of concatenated sequences for isolates with complete MLST profiles. A set of STs that represented supergroups A (ST-1 and ST-19), B (ST-9 and ST-41), D (ST-35), and F (ST-62) and a set of STs that had been previously isolated from Orthoptera hosts (id-24 (ST-21), id-25 (ST-32), id-1694 (ST-440), id-1703 (ST-448), and id-1707 (ST452)) were added to the ML tree reconstruction. Sequence alignments were generated in the MUSCLE software [69], and a nucleotide substitution model for each dataset was chosen by means of MEGA 6; statistical branch support was based on 1000 bootstrap iterations.

To expand the *Wolbachia* genetic diversity analysis, we created a dataset that included additional MLST profiles. The MLST profiles were chosen according to allele identity toward variants observed in Acrididae isolates (‘one allele criterion’, see details in refs. [46,62]. Briefly, we took an ST from the Public Databases for Molecular Typing and Microbial Genome Diversity (PubMLST) [70] if it contained the same allele as any of the loci found in our study. Because the number of alleles in our study was unique, we also included STs with the most closely related alleles. To present the phylogenetic relationships of profiles in this dataset, we reconstructed an unrooted phylogenetic network in SplitsTree4 [71] using the neighbour-net method [72]. In addition, we conducted a comprehensive phylogenetic analysis of the data on *Wolbachia* diversity discovered in *Ch. parallelus* [44] and in the above-mentioned dataset. We retrieved alleles from GenBank, assembled concatenated sequences, aligned the sequences, excluded redundant parts, and reconstructed the unrooted phylogenetic network. 

## 5. Conclusions

Many Acrididae species harbour Wolbachia symbionts. Nonetheless, Wolbachia ge-netic diversity is rather low among these hosts: (i) there are strains of only supergroups B and F, (ii) genetic variation is narrow within each supergroup. These data indicate mas-sive Wolbachia horizontal transmission among Acrididae hosts. Specific content of Wolbachia alleles in Acrididae hosts can be used for identifying route(s) and mechanism(s) of Wolbachia horizontal transmission.

## Figures and Tables

**Table 1:** The Acrididae specimens and *Wolbachia* infection.

Subfamily	Species	Region and Year of Collection	No. of Infected Specimens/Total
Gomphocerinae	*Arcyptera* (*Arcyptera*) *fusca* (Pallas, 1773)	Russia, Altai Mts, 2017	2/2
	*Arcyptera* (*Pararcyptera*) *microptera* (Fischer von Waldheim, 1833)	Russia, Altai Mts, 2017	0/5
	*Chorthippus* (*Chorthippus*) *albomarginatus* (De Geer, 1773)	Russia, Irkutsk region, 2016	10/10
	*Chorthippus* (*Glyptobothrus*) *apricarius* (Linnaeus, 1758)	Russia, Irkutsk region, 2016	7/7
	*Chorthippus* (*Glyptobothrus*) *biguttulus* (Linnaeus, 1758)	East Kazakhstan, 2007	0/6
	-‘’-	Russia, Novosibirsk region, 2017	174/198
	-‘’-	Russia, Irkutsk region	4/6
	-‘’-	Russia, Altai Mts, 2015	5/5
	*Chorthippus* (*Altichorthippus*) *intermedius* (Bey-Bienko, 1926)	Russia, Altai Mts, 2003	0/8
	*Chorthippus* (*Glyptobothrus*) *mollis* (Charpentier, 1825)	Russia, Altai Mts, 2003	2/5
	-‘’-	Turkey, 2003	0/9
	*Chorthippus fallax* (Zubovski, 1900)	Russia, Novosibirsk region, 2017	16/17
	*Chorthippus hammarstroemi* (Miram, 1907)	Russia, Altai Mts, 2003	0/5
	*Dociostaurus* (*Kazakia*) *brevicollis* (Eversmann, 1848)	East Kazakhstan, 2007	3/7
	*Dociostaurus* (*Kazakia*) *tartarus* (Stshelkanovtzev, 1921)	East Kazakhstan, 2007	0/5
	*Eclipophleps glacialis* (Bey-Bienko, 1933)	Russia, Altai Mts, 2003	2/8
	*Eremippus simplex* (Eversmann, 1859)	East Kazakhstan, 2007	0/2
	*Euthystira brachyptera* (Ocskay, 1826)	Russia, Altai Mts, 2003	0/2
	*Gomphocerippus rufus* (Linnaeus, 1758)	Russia, Novosibirsk region, 2017	1/7
	*Megaulacobothrus aethalinus* (Zubovski, 1899)	Russia, Altai Mts, 2003	0/6
	*Omocestus* (*Omocestus*) *viridulus* (Linnaeus, 1758)	Russia, Novosibirsk region, 2009	3/4
	-‘’-	Russia, Altai Mts, 2017	1/1
	*Omocestus* (*Omocestus*) *haemorrhoidalis* (Charpentier, 1825)	Russia, Altai Mts, 2017	1/2
	*Podismopsis altaica* (Zubovski, 1900)	Russia, Altai Mts, 2003	3/4
	*Podismopsis genicularibus* (Shiraki, 1910)	Russia, Sakhalin Is., 2010	2/3
	*Pseudochorthippus montanus* (Charpentier, 1825)	Russia, Novosibirsk region, 2017	40/50
	*Pseudochorthippus parallelus* (Zetterstedt, 1821)	Russia, Novosibirsk region, 2017	5/5
	*Stauroderus scalaris* (Fischer von Waldheim, 1846)	Russia, Altai Mts, 2017	8/8
	*Stenobothrus eurasius* (Zubovski, 1898)	Russia, Altai Mts, 2003	0/2
Oedipodinae	*Bryodema gebleri* (Fischer von Waldheim, 1836)	Russia, Altai Mts, 2003	2/3
	-‘’-	Russia, Altai Mts, 2017	2/2
	*Bryodema tuberculata* (Fabricius, 1775)	Russia, Altai Mts, 2003	0/3
	*Locusta migratoria* (Linnaeus, 1758)	Central Kazakhstan, 2007	0/4
	*Oedaleus decorus* (Germar, 1825)	Russia, Altai Mts, 2017	1/1
	-‘’-	East Kazakhstan, 2007	3/4
	-‘’-	Tadzhikistan, 2009	1/1
	*Psophus stridulus* (Linnaeus, 1758)	Russia, Altai Mts, 2003	0/1
	*Pyrgodera armata* (Fischer von Waldheim, 1846)	East Kazakhstan, 2007	0/4
Podisminae	*Anapodisma miramae* (Dovnar-Zapolskij, 1932)	Russia, Maritima region of Far East, 2008	1/1
	*Bohemanella frigida* (Boheman, 1846)	Russia, Altai Mts, 2003	1/3
	*Ognevia longipennis* (Shiraki, 1910)	Japan, Hokkaido, 2005	4/4
	-‘’-	Russia, Altai Mts, Edigan, 2003	5/5
	*Podisma pedestris* (Linnaeus, 1758)	Russia, Altai Mts, 2003	5/5
	-‘’-	Russia, Altai Mts, 2016	1/4
	*Podisma kanoi* (Storozhenko, 1994)	Japan, Honshu, 2005	1/1
	*Podisma sapporensis* (Shiraki, 1910)	Japan, Hokkaido Is, Tanno town vicinities, 2005	5/5
	-‘’-	Japan, Hokkaido Akan town vicinities, 2005	5/5
	-‘’-	Japan, Hokkaido, Yotei Mt., 2005	5/5
	-‘’-	Japan, Hokkaido, Disengen Mt., 2005	5/5
	-‘’-	Japan, Hokkaido, Naganuma town vicinities, 2005	5/5
	-‘’-	Japan, Hokkaido, Teine Mt., 2005	10/10
	-‘’-	Japan, Japan, Hokkaido, Shimokawa town vicinities, 2005	3/3
	*Podisma tyatiensis* (Bugrov & Sergeev, 1997)	Russia, Kuril Arch., Kunashir Is, 2001	1/1
	*Prumna littoralis* (Tarbinsky, 1932)	Russia, Maritima region of Far East, 2008	1/1
	*Prumna primnoa* (Motschulsky, 1846)	Russia, Sakhalin Is, 2010	5/5
	*Prumna ussuriensis* (Tarbinsky, 1930)	Russia, Maritima region of Far East, 2008	1/5
	*Sinopodisma punctata* (Mishchenko, 1954)	Japan, Ryukyu Arch., Ishigaki Is, 2005	0/3

**Table 2:** *Wolbachia* MLST profiles of the analysed Acrididae isolates.

Isolate	Species (Region, Year)	Supergroup	gatB	coxA	hcpA	ftsZ	fbpA	Sequ-Ence Type *
i-1	*Arcyptera fusca* (Altai, 2017)	F	~73 ** (MZ816480)	~63 (MZ816523)	~261 (MZ816567)	No ***	410 (MZ816669)	not full
i-2	*Chorthippus albomarginatus* (Irkutsk, 2016)	B	134 (MZ816445)	168 (MZ816488)	~6 (MZ816532)	106 (MZ816581)	197 (MZ816634)	h^ST^-1
i-3	*Ch*. *Apricarius* (Irkutsk, 2016)	B	134 (MZ816446)	14 (MZ816489)	~6 (MZ816533)	106 (MZ816582)	197 (MZ816635)	h^ST^-2
i-4	*Ch*. *Biguttulus* (Irkutsk, 2016)	B	9 (MZ816447)	133 (MZ816490)	~6 (MZ816534)	106 (MZ816583)	197 (MZ816636)	h^ST^-3
i-5	*Ch*. *Biguttulus* (Novosibirsk, 2017)	B	9 (MZ816448)	133 (MZ816491)	6 (MZ816535)	106 (MZ816584)	197 (MZ816637)	h^ST^-4
i-6	*Ch*. *Biguttulus* (Altai, 2015)	B	9 (MZ816481)	133 (MZ816524)	? ****	?	?	not full
i-7	*Ch*. *Mollis* (Altai, 2003)	B	9 (MZ816449)	133 (MZ816492)	6 (MZ816536)	106 (MZ816585)	197 (MZ816638)	h^ST^-4
i-8	*Ch*. *Fallax* (Novosibirsk, 2017)	B	9 (MZ816450)	133 (MZ816493)	6 (MZ816537)	106 (MZ816586)	197 (MZ816639)	h^ST^-4
i-9	*Dociostaurus brevicollis* (Kazakhstan, 2007)	F	~73 (MZ816451)	~63 (MZ816494)	~261 (MZ816538)	~269 (MZ816587)	410 (MZ816640)	h^ST^-5
i-10	*Eclipophleps glacialis* (G, Altai, 2003)	B	9 (MZ816482)	133 (MZ816525)	~6R (MZ816568)	No	~4 (MZ816670)	not full
i-11	*Gomphocerippus rufus* (G, Novosibirsk, 2017)	B	9 (MZ816452)	133 (MZ816495)	6 (MZ816539)	106 (MZ816588)	197 (MZ816641)	h^ST^-4
i-12	*Omocestus viridulus* (Novosibirsk, 2009)	B	9 (MZ816453)	133 (MZ816496)	6 (MZ816540)	106 (MZ816589)	197 (MZ816642)	h^ST^-4
i-13	*Om*. *Viridulus* (Altai, 2017)	B	134 (MZ816454)	168 (MZ816497)	~6 (MZ816541)	106 (MZ816590)	197 (MZ816643)	h^ST^-1
i-14	*Om*. *Haemorrhoidalis* (Altai, 2017)	B	134 (MZ816455)	14 (MZ816498)	~6 (MZ816542)	106 (MZ816591)	197 (MZ816644)	h^ST^-2
i-15	*Podismopsis altaica* (Altai, 2003)	F	No	~63 (MZ816526)	~325 (MZ816569)	?	?	not full
i-16	*Podismopsis genicularibus* (Sakhalin Is., 2010)	B–A	134 (MZ816456)	14 (MZ816499)	~6 (MZ816543)	226 (MZ816592)	197 (MZ816645)	h^ST^-6
i-17	*Pseudochorthippus montanus* (Novosibirsk, 2017)	B	9 (MZ816457)	133 (MZ816500)	6 (MZ816544)	106 (MZ816593)	197 (MZ816646)	h^ST^-4
i-18	*Ps*. *Montanus* (Novosibirsk, 2017)	B	9 (MZ816483)	133 (MZ816527)	6 (MZ816570)	?	?	not full
i-19	*Ps*. *Parallelus* (Novosibirsk, 2017)	B	9 (MZ816458)	133 (MZ816501)	6 (MZ816545)	106 (MZ816594)	197 (MZ816647)	h^ST^-4
i-20	*Stauroderus scalaris* (Altai, 2017)	B	9 (MZ816459)	133 (MZ816502)	6 (MZ816546)	106 (MZ816595)	197 (MZ816648)	h^ST^-4
i-21	*Bryodema gebleri* (Altai, 2003)	F	~73 (MZ816460)	~63 (MZ816503)	~261 (MZ816547)	~205 (MZ816596)	410 (MZ816649)	h^ST^-7
i-22	*Bryodema gebleri* (Altai, 2017)	F	~73 (MZ816461)	~63 (MZ816504)	~261 (MZ816548)	~205 (MZ816597)	410 (MZ816650)	h^ST^-7
i-23	*Oedaleus decorus* (Altai, 2017)	F	~73 (MZ816462)	~63 (MZ816505)	~35 (MZ816549)	~205 (MZ816598)	410 (MZ816651)	h^ST^-8
i-24	*Oe*. *Decorus* (Kazakhstan, 2006)	F	~243 (MZ816484)	~63RK (MZ816528)	~261 (MZ816571)	~205R (MZ816616)	410 (MZ816671)	N, full
i-25	*Oe*. *Decorus* (Tajikistan, 2009)	F	~243Y (MZ816485)	~30YR (MZ816529)	~261Y (MZ816572)	~205 (MZ816617)	410 (MZ816672)	N, full
i-26	*Anapodisma miramae* (Far East, Russia, 2008 )	B	39 (MZ816463)	14 (MZ816506)	40 (MZ816550)	7 (MZ816599)	197 (MZ816652)	ST299
i-27	*Bohemanella frigida* (Altai 2003)	B	9 (MZ816464)	9 (MZ816507)	6 (MZ816551)	106 (MZ816600)	197 (MZ816653)	h^ST^-9
i-28	*Ognevia longipennis* (Japan, 2005)	B	9 (MZ816465)	133 (MZ816508)	6 (MZ816552)	106 (MZ816601)	197 (MZ816654)	h^ST^-4
i-29	*Og*. *Longipennis* (Altai, 2003)	B	9 (MZ816466)	133 (MZ816509)	6 (MZ816553)	106 (MZ816602)	197 (MZ816655)	h^ST^-4
i-30	*Podisma pedestris* (Altai, 2003)	B	9 (MZ816467)	133 (MZ816510)	6 (MZ816554)	106 (MZ816603)	197 (MZ816656)	h^ST^-4
i-31	*P. pedestris* (Altai, 2016)	B	9 (MZ816468)	133 (MZ816511)	6 (MZ816555)	106 (MZ816604)	197 (MZ816657)	h^ST^-4
i-32	*Prumna littoralis* (Far East, Russia, 2008)	B	9 (MZ816469)	133 (MZ816512)	6 (MZ816556)	106 (MZ816605)	197 (MZ816658)	h^ST^-4
i-33	*Pr*. *Primnoa* (Sakhalin, 2010)	B	9 (MZ816470)	133 (MZ816513)	6 (MZ816557)	106 (MZ816606)	197 (MZ816659)	h^ST^-4
i-34	*Pr*. *Primnoa* (Sakhalin Is., 2010)	B	188 (MZ816471)	224 (MZ816514)	~6 (MZ816558)	20 (MZ816607)	25 (MZ816660)	h^ST^-10
i-35	*Pr*. *Ussuriensis* (Far East, Russia, 2008)	B	9 (MZ816472)	133 (MZ816515)	6 (MZ816559)	106 (MZ816608)	197 (MZ816661)	h^ST^-4
i-36	*Podisma kanoi* (Honshu, Japan, 2005)	B	9 (MZ816473)	133 (MZ816516)	6 (MZ816560)	106 (MZ816609)	197 (MZ816662)	h^ST^-4
i-37	*P. sapporensis* (Japan, Tanno, 2005)	B	9 (MZ816474)	14 (MZ816517)	6 (MZ816561)	106 (MZ816610)	197 (MZ816663)	h^ST^-11
i-38	*P. sapporensis* (Japan, Akan, 2005)	B	9 (MZ816475)	14 (MZ816518)	6 (MZ816562)	106 (MZ816611)	197 (MZ816664)	h^ST^-11
i-39	*P. sapporensis* (Japan, Yotei, 2005)	B	9 (MZ816476)	14 (MZ816519)	6 (MZ816563)	106 (MZ816612)	197 (MZ816665)	h^ST^-11
i-40	*P. sapporensis* (Japan, Disengen, 2005)	B	9 (MZ816486)	14 (MZ816530)	6 (MZ816573)	Mix ***** (MZ816618-MZ816632)	197 (MZ816673)	Mix
i-41	*P. sapporensis* (Japan, Naganuma, 2005)	B	9 (MZ816477)	14 (MZ816520)	6 (MZ816564)	106 (MZ816613)	197 (MZ816666)	h^ST^-11
i-42	*P. sapporensis* (Japan, Teine, 2005)	B	9 (MZ816487)	73 (MZ816531)	Mix (MZ816574-MZ816580)	106 (MZ816633)	Mix (MZ816674-MZ816686)	Mix
i-43	*P. sapporensis* (Japan, Shimokawa, 2005)	B	9 (MZ816478)	14 (MZ816521)	6 (MZ816565)	106 (MZ816614)	197 (MZ816667)	h^ST^-11
i-44	*P. tyatiensis* (Kunashir Is., Russia, 2001)	B	9 (MZ816479)	14 (MZ816522)	6 (MZ816566)	106 (MZ816615)	197 (MZ816668)	h^ST^-11

* ST numbers according to the PubMLST database or haplotype numbers according to this study or short comments are provided; ** the ‘~ number’ refers to the most closely related alleles according to the PubMLST database; *** no PCR product; **** multiple double chromatogram peaks; ***** mix: a DNA sample that yielded multiple double chromatogram peaks was cloned and sequenced.

## Data Availability

Not applicable.

## Supplementary Materials

The following supporting information can be downloaded at: https://www.mdpi.com/article/10.3390/ijms23020853/s1.
