# Supplementary figures and images for: Narrow Genetic Diversity of Wolbachia Symbionts in Acrididae Grasshopper Hosts (Insecta, Orthoptera)
Source: Int J Mol Sci. 2022 Jan 13;23(2):853. doi: 10.3390/ijms23020853 (PMC8775660; doi:10.3390/ijms23020853)

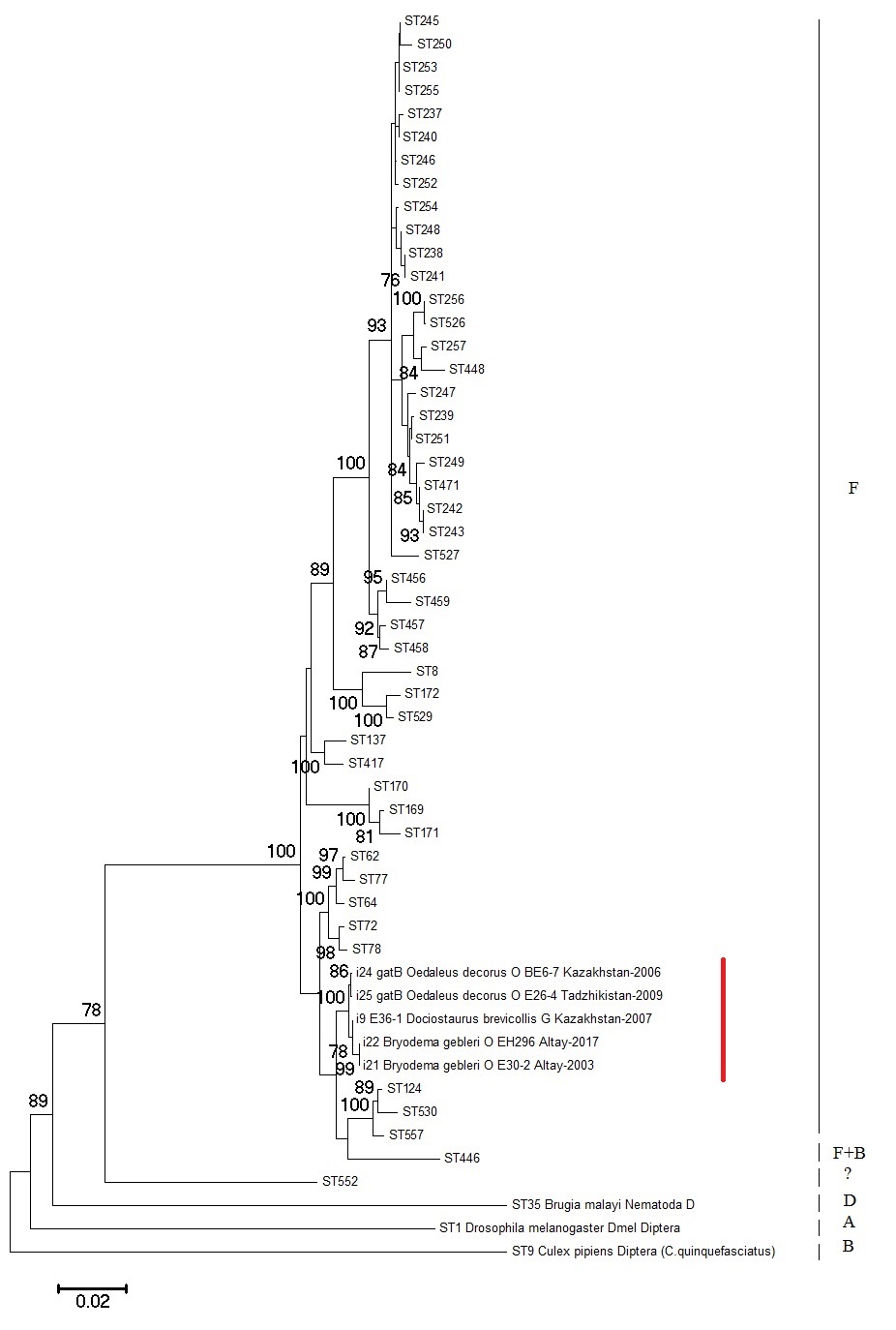

Supplement: Supplementary file 1 [file ijms-23-00853-s001.zip › FigS1_treeF_MLST.jpg]
